# Supplementary figures and images for: Correlation between Retinal Vascularization and Disease Aggressiveness in Amyotrophic Lateral Sclerosis
Source: Biomedicines. 2022 Sep 25;10(10):2390. doi: 10.3390/biomedicines10102390 (PMC9598742; doi:10.3390/biomedicines10102390)

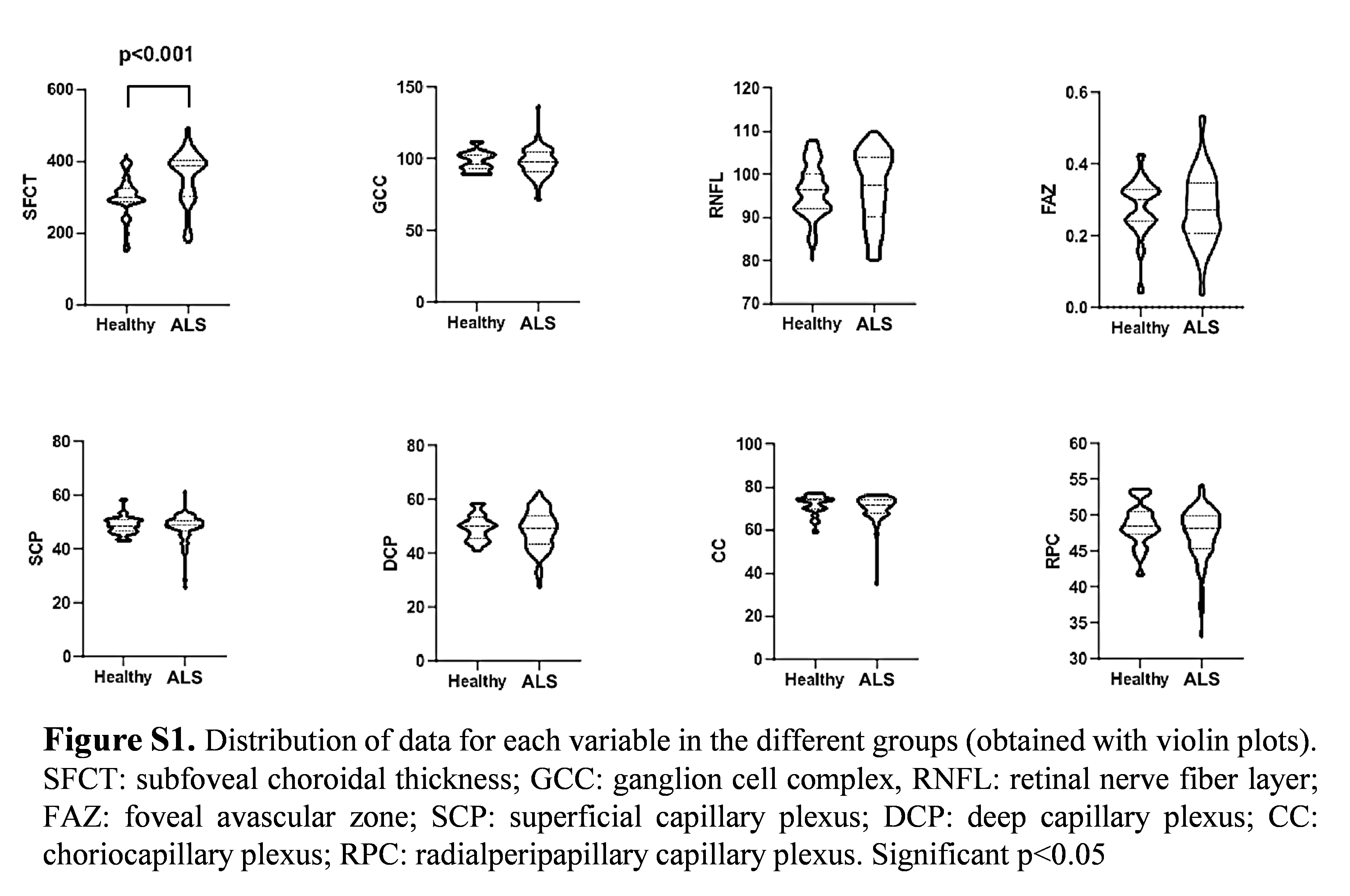

Supplement: Supplementary file 1 [file biomedicines-10-02390-s001.zip › Figure S1.tif]

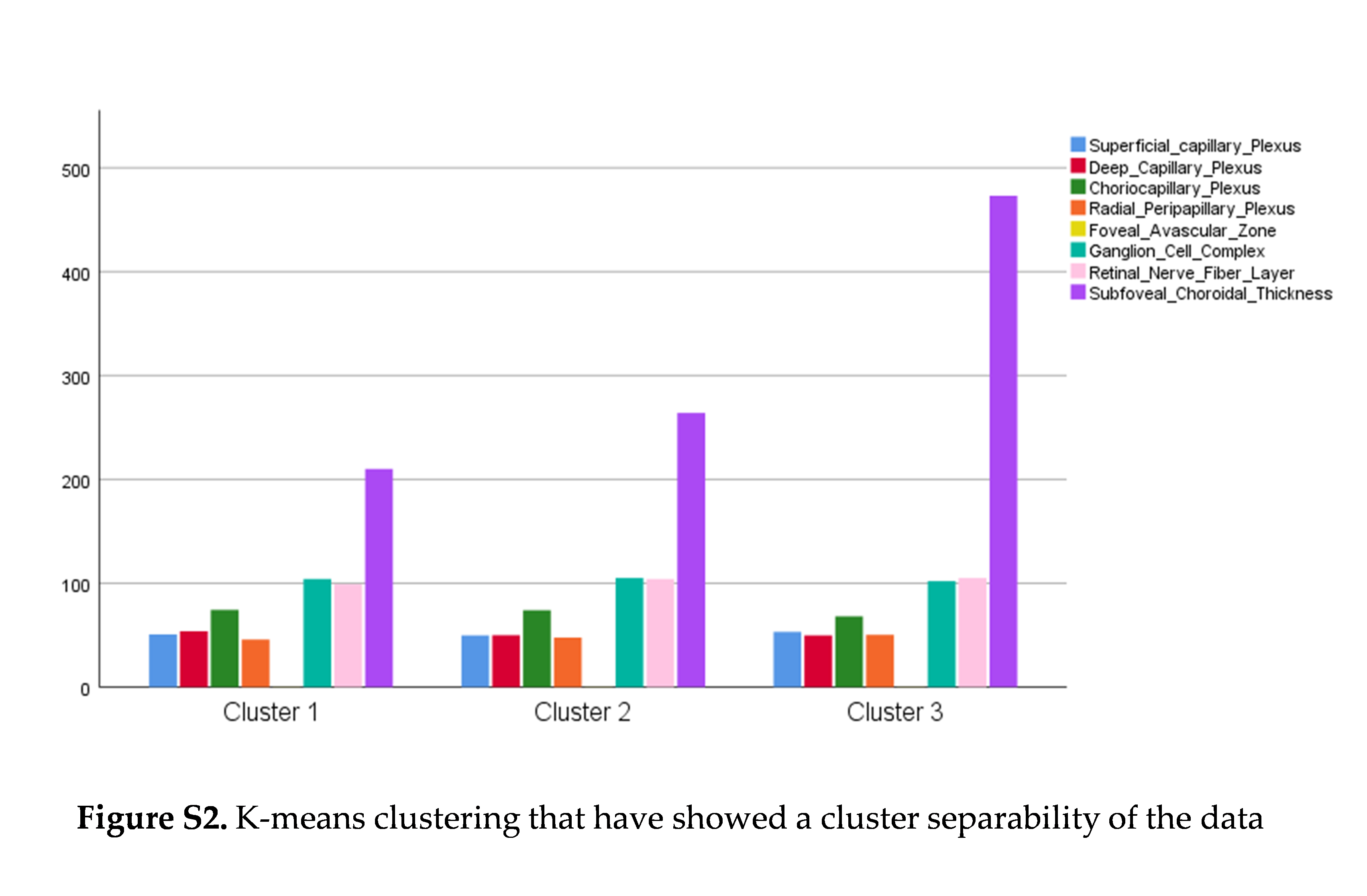

Supplement: Supplementary file 1 [file biomedicines-10-02390-s001.zip › Figure S2.tif]
